# The Hedgehog Pathway Promotes Monocytes Infiltration Through CCL20–CCR6 Axis in Hepatocellular Carcinoma

**DOI:** 10.1111/jcmm.70824

**Published:** 2025-09-05

**Authors:** Pei‐Han Chu, Yu‐Fu Hsu, Chen‐Yi Chang, Chuen‐Miin Leu, Kuo‐Hsin Chen, Chiung‐Fang Chang, Ping‐Hui Tseng

**Affiliations:** ^1^ School of Life Science, Institute of Biochemistry and Molecular Biology National Yang Ming Chiao Tung University Taipei Taiwan; ^2^ School of Life Science, Institute of Microbiology and Immunology National Yang Ming Chiao Tung University Taipei Taiwan; ^3^ Division of General Surgery, Department of Surgery Far Eastern Memorial Hospital New Taipei City Taiwan; ^4^ Division of Electrical Engineering Yuan Ze University Taoyuan Taiwan; ^5^ Department of Medical Research Far Eastern Memorial Hospital New Taipei City Taiwan

**Keywords:** Hedgehog pathway, hepatocellular carcinoma, monocytes and CCL20

## Abstract

Hepatocellular carcinoma (HCC) is one of the leading cancers worldwide, and its development is strongly associated with the tumour microenvironment, particularly fibrosis and chronic inflammation. This study aims to investigate the role of the Hedgehog (Hh) pathway, a key signalling pathway in HCC progression, in the interaction between HCC cells and monocytes, which are central players in inflammation. Using a transwell migration assay, GLI1, the downstream transcriptional effector of the Hh pathway in HCC cells, was found to promote the migration of THP‐1 monocyte cells. Among the cytokines regulated by the Hh pathway in HCC cells, CCL20 was identified as a crucial factor that interacts with CCR6 in THP‐1 cells to facilitate migration. Next, using a luciferase reporter assay and chromatin immunoprecipitation, GLI1 binding sites within the *CCL20* promoter region were confirmed. In a xenograft tumour mouse model, tumour growth and monocyte infiltration were inhibited in *GLI1* or *CCL20* knockout PLC5 cells. Moreover, mRNA expressions of *GLI1* and *CCL20* were positively correlated in clinical samples, with patients exhibiting high CCL20 expression showing poorer overall survival. Overall, our findings highlight that the Hh pathway in HCC contributes to monocyte infiltration via the CCL20–CCR6 axis, providing potential insights for future therapeutic strategies.

## 1 Introduction

Hepatocellular carcinoma (HCC) is one of the most common cancers worldwide [1]. Major risk factors for HCC include hepatitis B virus (HBV) and hepatitis C virus (HCV) infections, alcohol and tobacco use, and non‐alcoholic steatohepatitis associated with metabolic syndrome or diabetes mellitus. These factors drive chronic inflammation and liver damage [2, 3]. Activation of signalling pathways such as Wnt–TGFβ, Ras‐MAPKs and PI3K/Akt promotes cellular proliferation and contributes to HCC tumourigenesis [4, 5, 6]. As an inflammation‐associated cancer, the JAK‐STAT3 and NF‐κB pathways, the key inflammatory signalling, are critical for HCC progression [7, 8, 9].

The Hedgehog (Hh) signalling pathway has also been shown to play a role in various aspects of HCC development and progression [10, 11]. In canonical Hh signalling, the pathway is activated when one of three Hh protein ligands, Sonic Hedgehog (Shh), Indian Hedgehog (Ihh) and Desert Hedgehog (Dhh), binds to the Patched (PTCH) receptor on the cell surface. Under normal conditions, PTCH represses the activity of Smoothened (SMO), a G‐protein coupled receptor [12]. Upon ligand binding, SMO is released from PTCH inhibition, triggering a signalling cascade that activates the GLI family of transcription factors (GLI1, GLI2 and GLI3), to promote the transcription of target genes [13, 14]. Additionally, non‐canonical Hh signalling can occur independently of PTCH, SMO or GLI, but does not follow the canonical route [15]. Abnormal activation of the Hh pathway in the liver promotes regeneration, vascular remodelling, fibrosis and HCC, and is associated with lower survival and higher recurrence rates in HCC patients [16, 17, 18].

The tumour microenvironment, composed of immune cells, inflammatory factors and non‐immune components, plays a critical role in HCC progression by promoting tumour growth, metastasis and immune evasion [19]. During chronic inflammation, various inflammatory factors, such as interleukin 6 (IL6), tumour necrosis factor (TNF) and interferon (IFN), are released, and immune cells, including T lymphocytes, macrophages, neutrophils, and dendritic cells, accumulate within the tumour and surrounding tissues [20]. Non‐immune components, including hepatic stellate cells (HSCs), cancer‐associated fibroblasts (CAFs), blood vessels, lymphatic endothelial cells and the extracellular matrix (ECM), interact with immune cells to mediate tissue remodelling and disrupt normal liver function, contributing to fibrosis, cirrhosis and angiogenesis [21].

The Hh pathway has been shown to modulate the tumour microenvironment by inducing fibrosis and promoting angiogenesis, thereby supporting HCC progression [22, 23]. However, the interaction between the Hh pathway and inflammatory microenvironments in HCC remains poorly understood. Given evidence from various cancers indicating that inflammatory factors, such as IL6, TNF or IFN, are regulated by GLI1, and that the Hh pathway is involved in cancer immunity [24], we hypothesise that the Hh pathway in HCC cells may mediate the production of cytokines or chemokines that promote monocyte infiltration, thereby modulating the tumour microenvironment to support tumour growth and metastasis.

## 2 Materials and Methods

### 2.1 Ethics Statement

The use of human samples and animals was in accordance with national approved law (Human Subjects Research Act and Animal Protection Act) and institutional approved guidelines. Archived human HCC tumour samples were obtained from the Taiwan Liver Cancer Network (TLCN) and approved by the Institutional Review Board of National Yang‐Ming Chiao Tung University (YM103007E). Human samples were collected under the approval of the Institutional Review Board of Far Eastern Memorial Hospital (113015‐E, 110239‐F). All animal studies were performed according to the animal study protocol approved by the Institutional Animal Care and Use Committee (IACUC) of National Yang Ming Chiao Tung University (IACUC #1121112) and the recommendation of ‘A Guidebook for Care and Use of Laboratory Animals’ (Third Edition). All efforts were made to minimise animal discomfort and suffering.

### 2.2 Reagents and Antibodies

The lentivirus‐based shRNA expression plasmids, including pLKO.1‐shGLI1 (TRCN0000020485 and TRCN0000232062) for human GLI1, pLKO.1‐shCCL20 (TRCN0000057964) for human CCL20, pLKO.1‐shCSF2 (TRCN0000058428) for human CSF2, pLKO.1‐shIL6 (TRCN0000059206) for human IL6, pLKO.1‐shIL17A (TRCN0000048728) for human IL17A, pLKO.1‐shCCR6 (TRCN0000008205) for human CCR6, pLKO.1‐shLuc (TRCN0000072243) for luciferase, and the VSV‐G‐pseudotyped lentivirus packaging plasmids, pCMVΔ8.91 and pMD.G, were obtained from National RNAi Core Facility (Institute of Molecular Biology/Genomics Research Center, Academia Sinica, Taiwan). pRL‐TK‐Renilla luciferase and pGL3‐Control Vector used for the luciferase reporter assay were from Promega (WI, USA). All constructs were confirmed by DNA sequencing. Anti‐F4/80 antibody (GTX26640) was from GeneTex (CA, USA), anti‐CSF‐1‐R (CD115) antibody (EPR23529‐26) was from Abcam (Cambridge, UK) and anti‐Ly‐6C antibody (128002) was from Biolegend (CA, USA).

### 2.3 Cell Culture and Treatment

Human HEK293T and human PLC/PRF5 (PLC5) cells were obtained from Bioresource Collection and Research Center (BCRC, Taiwan), and human Huh7 cells were from the Japanese Collection of Research Bioresources (JCRB). The cells were grown in Dulbecco's modified Eagle's medium (DMEM) containing 10% fetal bovine serum (FBS), 1 mM sodium pyruvate and 1% (v/v) penicillin–streptomycin solution. DMEM, sodium pyruvate and penicillin–streptomycin solution were obtained from Biological Industries, and FBS was purchased from ThermoFisher (MA, USA). For collecting conditional medium from cultured HCC cells, 5 × 10^5^ cells were seeded in the 10 cm plate for 24 h. After changing to fresh 10% FBS containing medium, the cells were incubated for 24 h. The conditional medium (CM) was harvested, collected, filtrated by a 0.45 μm filter, and used for experiments immediately. For treatment with Shh ligand (1 μg/mL), Recombinant Human Sonic Hedgehog/SHH Protein (aa 1–197, His Tag) was purchased from Sino Biological (10372‐H08H1, Beijing, China) and dissolved in phosphate‐buffered saline (PBS).

A portion of tumour tissue was freshly processed to isolate primary human HCC cells, following a protocol similar to that in Cheung et al.'s study [25]. The HCC primary cells were cultured in DMEM containing 20% FBS, 1 mM sodium pyruvate and 1% (v/v) penicillin–streptomycin solution. The HCC primary cells were treated with or without Hedgehog inhibitor HPI‐1 (Cayman, MI, USA) for 48 h and subjected to NGS analysis for whole gene expression profiling.

### 2.4 Migration Assay

Migration assay was set up by 8‐μm‐pore transwell insert (Jet Biofil, Guangzhou, China). 1 × 10^5^ THP‐1 cells were resuspended in the upper chamber of a transwell plate and fresh medium or conditioned medium (CM) was added to the lower chamber. Following incubation for 12 h, THP‐1 cells that migrated through the transwell membrane into the medium of the lower chamber were collected and counted using a haemocytometer.

### 2.5 Lentivirus Production and Infection

HEK293T cells were transfected with the lentivirus‐based constructs along with the packaging plasmids, pCMVΔ 8.91 and pMD.G using T‐pro NTRII (T‐Pro Biotechnology, Taiwan). Virus‐containing medium was collected at 24 and 48 h post‐transfection. Cells were infected with lentivirus‐containing medium at a multiplicity of infection (MOI) of 10–25 in the presence of 10 μg/mL polybrene (Sigma, NJ, USA). After 24 h, the virus‐containing medium was replaced with fresh medium containing 3 mg/mL puromycin (EMD Millipore, MA, USA). After cell growth was stable, cells were used in subsequent experiments.

### 2.6 RNA Isolation and Real‐Time Quantitative Polymerase Chain Reaction (RT‐qPCR)

Total RNA was isolated with TRIzol (ThermoFisher), and reverse transcribed to synthesise cDNA with 10 μM random hexamer (for RT of mRNA) by iScript cDNA Synthesis Kit (Bio‐Rad, CA, USA). Target mRNAs were quantified by RT‐qPCR with the StepOnePlus system (Applied Biosystems, MA, USA). The real‐time PCR parameters were as follows: hot start at 95°C for 1 min, 45 cycles of denaturing at 95°C for 10 s, annealing at 60°C for 5 s and extension at 72°C for 20 s. Primer sequences are available in Table S1. The relative level of target gene expressions was obtained by normalisation to the level of *GAPDH* mRNA expression.

### 2.7 Next Generation Sequencing (NGS) of Differential Gene Expressions

RNA was fragmented for library preparation using 200–500 ng of total RNA following the protocol provided in the TrueSeq Stranded mRNA Kit (Illumina, CA, USA). For sequencing, the prepared mRNA libraries were processed using the HiSeq 3000/4000 PE Cluster Kit and sequenced on the HiSeq 4000 platform (Illumina). Data mapping was performed using Bowtie 2, while gene expression quantification was conducted with RSEM. Differential gene expression analysis was carried out using the Ingenuity Pathway Analysis (IPA) software (Qiagen, CA, USA).

### 2.8 Luciferase Reporter Assay

As previously described [26], PLC5 cells were seeded onto a 6‐well plate at 3 × 10^5^ cells per well and co‐transfected with 5000 ng indicated luciferase reporter constructs using T‐pro NTRII. After transfection for 24 h, the transfected cells were seeded onto a 96‐well plate at 1 × 10^4^ cells per well and treated with or without Shh ligands for 12 h. The luciferase activity was monitored using the Dual‐Glo luciferase assay system and a luminometer. Renilla luciferase activity was normalised against firefly luciferase activities and presented as a percentage of inhibition.

### 2.9 Chromatin Immunoprecipitation (ChIP)

ChIP was performed as described previously [27]. Briefly, cells were cross‐linked with 1% formaldehyde for 10 min at room temperature and quenched by 125 mM glycine and lysed in lysis buffer (50 mM Tris–HCl pH 7.9, 1 mM EDTA pH 8.0, 0.1% SDS). Chromatin was fragmented by a Covaris (S220) Sonicator and sonicated chromatin was diluted in dilution buffer (50 mM HEPES pH 7.5, 1 mM EDTA, 150 mM NaCl, 1% TX100). The immuno‐complexes were precipitated with control IgG or Dynabeads Protein G (Invitrogen)‐conjugated antibody (against GLI1). Precipitated complexes were RNase A treated overnight at 65°C and proteins digested with proteinase K at 65°C for 1 h. After washing the beads with high salt buffer containing 20 mM Tris pH 8.1, 2 mM EDTA, 0.1% SDS, 1% TX100 and 500 mM NaCl, precipitated DNAs were purified by PCR purification kit (Qiagen) and analysed by PCR using indicated primers. Primer sequences are available in Table S1.

### 2.10 Generation of *GLI1* or *CCL20* Knockout Cell Lines

CRISPR/Cas9‐mediated *GLI1* or *CCL20* knockout in PLC5 cells was carried out following the protocol of Ran et al. [28]; PX459 [pSpCas9(BB)‐2A‐Puro V2.0] was obtained from Addgene (Plasmid #62988) [5]. The short guide RNAs (sgRNAs) against GLI1 or CCL20 were designed and cloned into the all‐in‐one PX459 vector. The sequences for gRNA are available in Table S1. PLC5 cells were transfected with either gRNA 1 or 2, using T‐pro NTRII, and selected with puromycin (3 μg/mL). Single clones were separated by dilution and seeded in 96‐well plates to derive two independent clonal cell populations (clone 1 and 2). Gene knockout was validated by Sanger sequencing and qPCR.

### 2.11 Mouse Xenograft Tumour Formation

For xenograft tumour model, 6‐week‐old male SCID mice were obtained from the National Laboratory Animal Center (Taipei, Taiwan). Each mouse was inoculated s.c. in the right dorsal flank with indicated cancer cells (5 × 10^5^) suspended in 50 μL PBS and equal volume of Matrigel (BD Biosciences, NJ, USA). Tumours were measured with Vernier callipers. Tumour volumes were calculated with the following formula: width^2^ × length × 0.52 [29]. Whenever tumour size reaches 1000 mm^3^ before the end of treatments, mice are sacrificed based on the Animal Care and Use Guidelines.

### 2.12 H&E and Immunohistochemical (IHC) Analysis

Tumour samples were processed for histology, embedded in paraffin, and sectioned at a thickness of 5 μm. For H&E staining, the sections were stained with haematoxylin and eosin for 10 min and 30 s, respectively. Immunohistochemistry was performed according to established protocols using F4/80, Ly6C and CD115 antibodies. The quantification of histology was performed by Image‐Pro Plus 5.0 (Leica, Wetzlar, Germany).

### 2.13 Study Subjects

The tumour tissues were collected from Stage I or II (based on The American Joint Committee on Cancer [AJCC] TNM system), HBV and HCV negative, without receiving chemotherapy or radiotherapy, and male patients in the Taiwan Liver Cancer Network (TLCN). The HCC samples were used for examining the expression of *GLI1* and *CCL20* mRNA clinically. In addition, primary HCC cells were obtained from small HCC tissue samples, either following surgical resection or from the Human Biobank at Far Eastern Memorial Hospital.

### 2.14 Statistical Analysis

Data was collected in Microsoft Excel and reported as mean ± SD. The comparison of means was performed by multiple *t*‐test, and the comparisons between different groups for each point will be performed by the one‐way analysis of variance (ANOVA) using GraphPad Prism (version 8.3.0, MA, USA). Correlation analysis was completed using the Spearman method with SPSS statistical software (IBM SPSS Statistics 20.0, NY, USA). All tests are two‐tailed, and a *p* value of less than 0.05 (*p < 0.05, **p < 0.01) was considered statistically significant.

## 3 Results

### 3.1 The Hedgehog Pathway Is Involved in HCC‐Mediated Promotion of Monocyte Migration and Macrophage Polarisation

**FIGURE 1:**
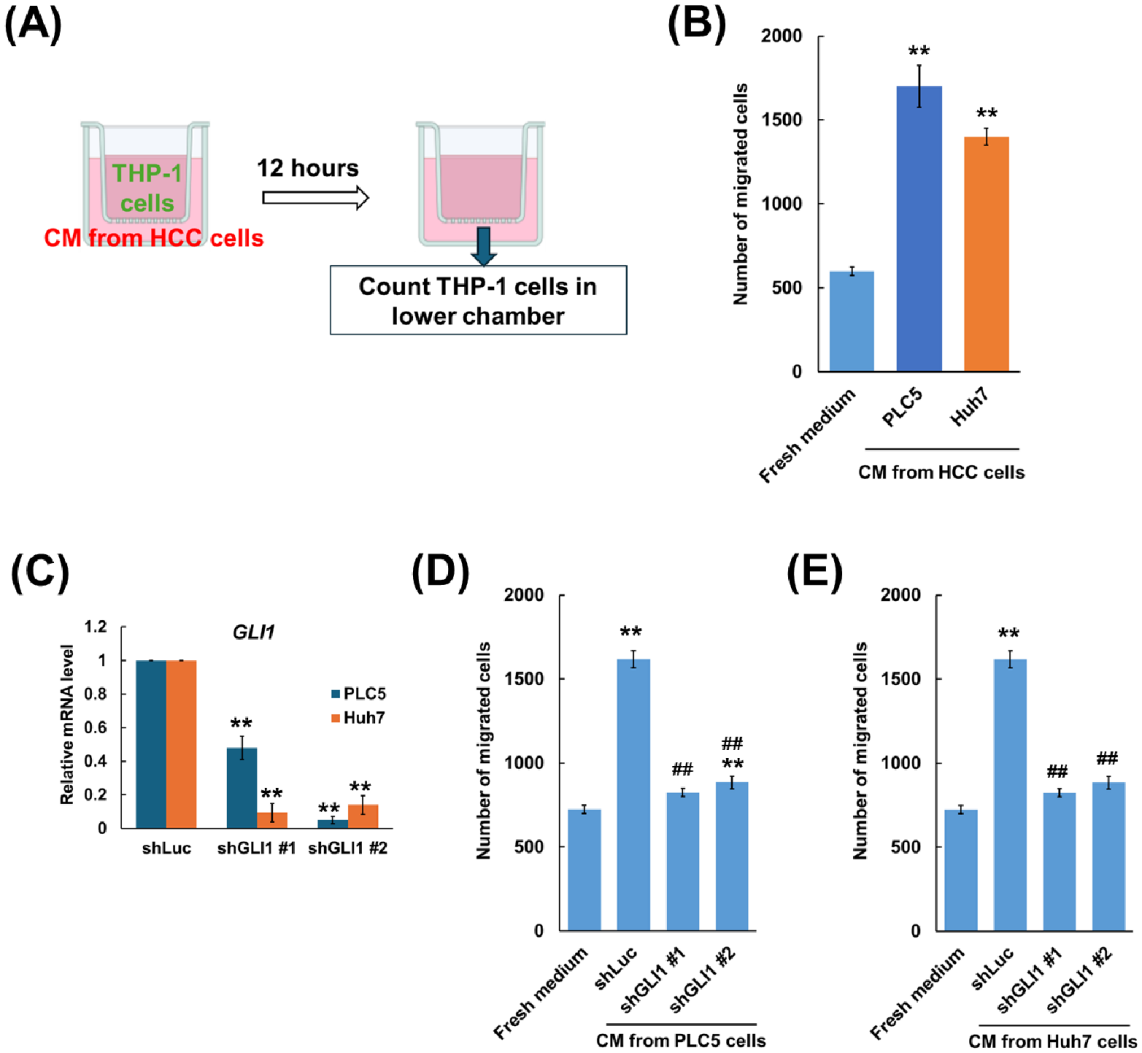
Hedgehog signalling pathway HCC is involved in promoting monocyte migration. (A) Schematic illustration of the transwell migration assay for THP‐1 cells. (B) Effects of conditional medium collected from PLC5 or Huh7 cell culture on THP‐1 cell migration. The number of migrated THP‐1 cells was counted, and fresh medium containing 10% FBS was served as the control. (C) qPCR analysis of GLI1 mRNA level. PLC5 or Huh7 cells were transduced with control or shGLI1, and the fold change of GLI1 mRNA was determined comparing with shLuc. THP‐1 cell migration was affected by conditional medium collected from (D) PLC5 or (E) Huh7 cell culture with or without GLI1 knockdown. Results are expressed as the mean ± SD of three separate experiments (**p < 0.01, vs. fresh medium or shLuc control; ^##^ *p* < 0.01, vs. conditional medium from shLuc cells).

We investigated monocyte migration mediated by HCC cells using a transwell assay. Our results showed that conditional medium collected from PLC5 or Huh7 HCC cells promoted the migration of THP‐1 monocyte cells (Figure 1A). To examine the role of the Hh pathway in the composition of the conditional medium from HCC cells, GLI1 expression, a downstream effector of the Hh pathway, was knocked down using shRNA targeting two distinct regions of *GLI1* mRNA in PLC5 and Huh7 cells, with successful knockdown confirmed by qPCR (Figure 1B). In the transwell assay, THP‐1 cell migration was reduced in the conditional medium collected from *GLI1*‐knockdown PLC5 or Huh7 cells compared with the medium from shLuc control cells (Figure 1C,D). These findings indicate that the Hh pathway is involved in regulating the production of cytokines or chemokines by HCC cells to promote monocyte migration.

### 3.2 The Hedgehog Pathway in HCC Cells Induced the Production of Inflammatory Cytokines or Chemokines

**FIGURE 2:**
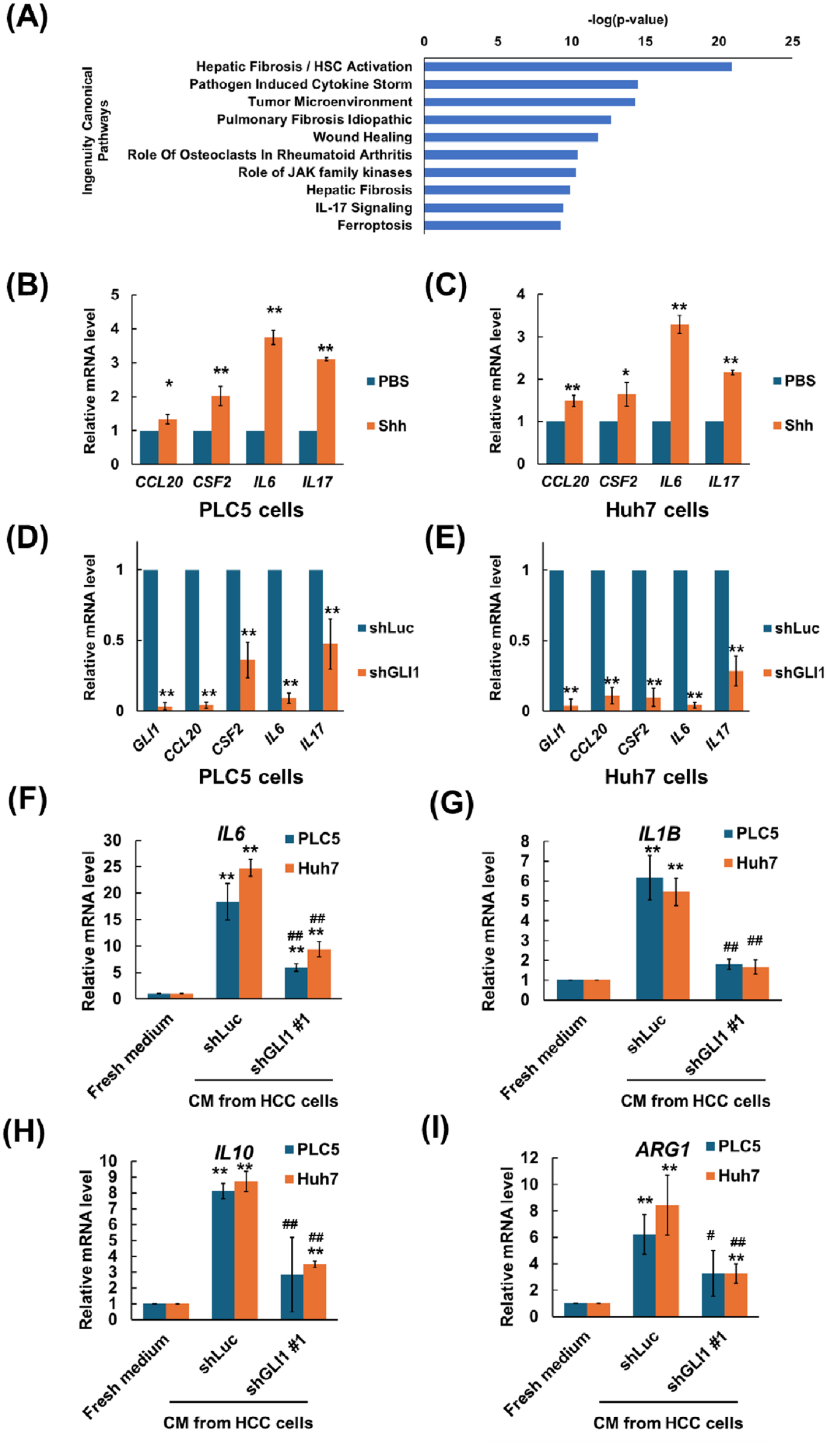
Hedgehog signalling pathway induced the production of inflammatory cytokines or chemokines in HCC cells. (A) GLI‐regulated DEGs in canonical signalling pathways. DEGs from five different primary HCC cells with or without the HPI‐1 treatment were subjected to the analysis of canonical signalling pathways. The top 10 enriched canonical signalling pathways were identified by Ingenuity Pathway Analysis. qPCR analysis of mRNA levels including CCL20, CSF2, IL6 and IL17 in (B) PLC5 and (C) Huh‐7 cells treated with DMSO or Shh ligand. Fold change of indicated mRNA was determined by comparing with DMSO. qPCR analysis of mRNA levels including CCL20, CSF2, IL6 and IL17 in (D) PLC5 and (E) Huh‐7 cells transduced with shLuc or shGLI1. Fold change of indicated mRNA was determined by comparing with PBS or shLuc cells. qPCR analysis of (F) IL6, (G) IL1B, (H) IL10 and (I) ARG1 mRNA levels for THP‐1 cell polarisation with incubation of conditional medium collected from PLC5 or Huh7 cell culture. Results are expressed as the mean ± SD of three separate experiments (**p < 0.01, vs. shLuc control or fresh medium; ^#^ *p* < 0.05, ^##^ *p* < 0.01, vs. conditional medium from shLuc cells).

Next, we analysed the genes regulated by the Hedgehog pathway. Using RNA‐seq analysis for five primary HCC cell lines derived from HCC patients, the differentially expressed genes (DEGs) in cells treated with HPI‐1, a Hh pathway inhibitor, were identified. These DEGs were further categorised into different cellular functions by Ingenuity Pathway Analysis. The pathogen‐induced cytokine storm signalling pathway and IL‐17 signalling pathway ranked among the top 10 pathways with a negative *Z*‐score and significant *p*‐value (Figure 2A). Genes in these pathways included CC chemokine ligand 20 (CCL20), IL6, colony‐stimulating factor‐2 (CSF2) and IL17 (Table S2). To validate these DEGs, mRNA expression of genes in the cytokine storm signalling pathway, including CCL20, CSF2, IL6 and IL17, were examined by qPCR. Upon treatment with Shh ligand (1 μg/mL) for 12 h to activate the Hh pathway in PLC5 or Huh7 cells, the mRNA levels of *CCL20*, *CSF2*, *IL6* and *IL17* showed a marginally increased expression (Figure 2B,C). Conversely, the expression levels of *CCL20*, *CSF2*, *IL6* and *IL17* mRNA were decreased in PLC5 or Huh7 cells with GLI1 knockdown compared with shLuc control cells (Figure 2D,E).

Since CCL20, CSF2, IL6 and IL17 have been identified as critical targets of Hh signalling in HCC, the correlation between the Hh pathway of HCC cells and macrophage polarisation was investigated. THP‐1 cells were incubated with fresh medium or conditional medium collected from control or *GLI1*‐knockdown PLC5 or Huh7 cells for 3 days, and the expression of marker genes for M1 and M2 macrophages was analysed by qPCR. The mRNA level of M1 markers, including *IL6* and *IL1B* and M2 markers, including *IL10* and *ARG1*, showed significant decreases in cells treated with GLI1‐knockdown conditioned medium compared with those treated with shLuc control medium (Figure 2F–I).

### 3.3 The Hedgehog Pathway in HCC Mediates Monocyte Migration Through CCL20–CCR6 Axis

**FIGURE 3:**
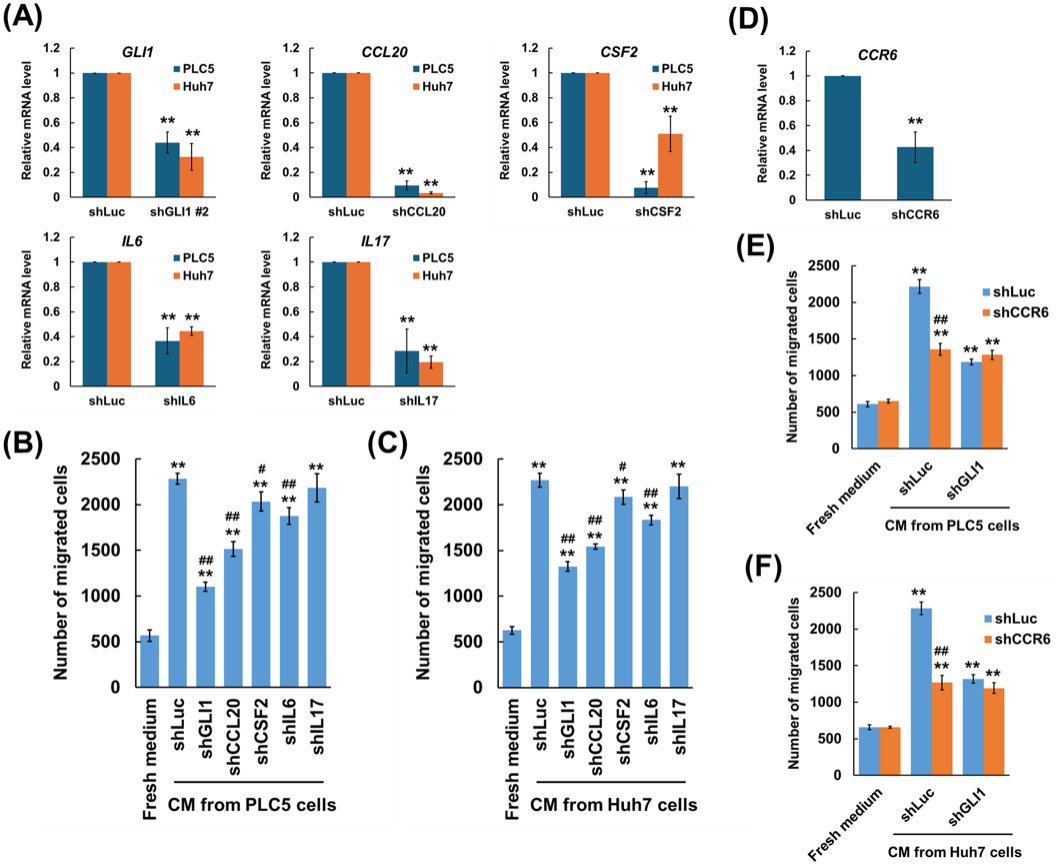
CCL20 secreted by HCC cells is involved in GLI1‐mediated promotion of monocyte migration through CCR6 in THP‐1 cells. (A) qPCR analysis of mRNA levels including GLI1, CCL20, CSF2, IL6 and IL17. PLC5 or Huh7 cells were transduced with indicated shRNA, and fold change of indicated mRNA was determined comparing with shLuc. THP‐1 cell migration affected by conditional medium collected from (B) PLC5 or (C) Huh7 cell culture with or without GLI1 knockdown. (D) qPCR analysis of CCR6 mRNA level. THP‐1 cells were transduced with control or shCCR6, and fold change of indicated mRNA was determined compared with shLuc. THP‐1 cell migration with or without CCR6 knockdown affected by conditional medium collected from (E) PLC5 or (F) Huh7 cell culture with or without GLI1 knockdown. Results are expressed as the mean ± SD of three separate experiments (**p < 0.01, vs. shLuc control or fresh medium; ^#^ *p* < 0.05, ^##^ *p* < 0.01, vs. conditional medium from shLuc cells).

To assess the involvement of CCL20, CSF2, IL6 and IL17 in monocyte migration, PLC5 and Huh7 cells were transduced with the indicated lentiviral shRNA, respectively, and successful gene knockdown was confirmed by qPCR (Figure 3A). In a transwell migration assay, conditional medium collected from *CCL20* knockdown PLC5 or Huh7 cells significantly reduced THP‐1 cell migration compared to shLuc control. In contrast, conditional medium from *CSF2* and *IL6* knockdown cells resulted in a marginal decrease, while medium from *IL17* knockdown cells showed no difference compared to shLuc controls (Figure 3B,C). These results suggest that CCL20 might be a key chemokine regulated by the Hh pathway in HCC to promote monocyte migration.

To further explore this, the role of chemokine receptor CC chemokine receptor 6 (CCR6), the unique receptor of CCL20, in monocytes was investigated. CCR6 expression in THP‐1 cells was knocked down using shRNA targeting CCR6 (Figure 3D). In a transwell assay, migration of *CCR6* knockdown THP‐1 cells was significantly decreased in response to conditional medium from control HCC cells (Figure 3E,F). However, no difference in migration was observed between control and *CCR6* knockdown THP‐1 cells when exposed to conditional medium from *GLI1* knockdown HCC cells (Figure 3E,F). These results indicate the association of GLI1‐regulated CCL20 in HCC cells and CCR6 in monocytes, demonstrating that the CCL20–CCR6 axis plays a role in HCC‐mediated monocyte migration.

**FIGURE 4:**
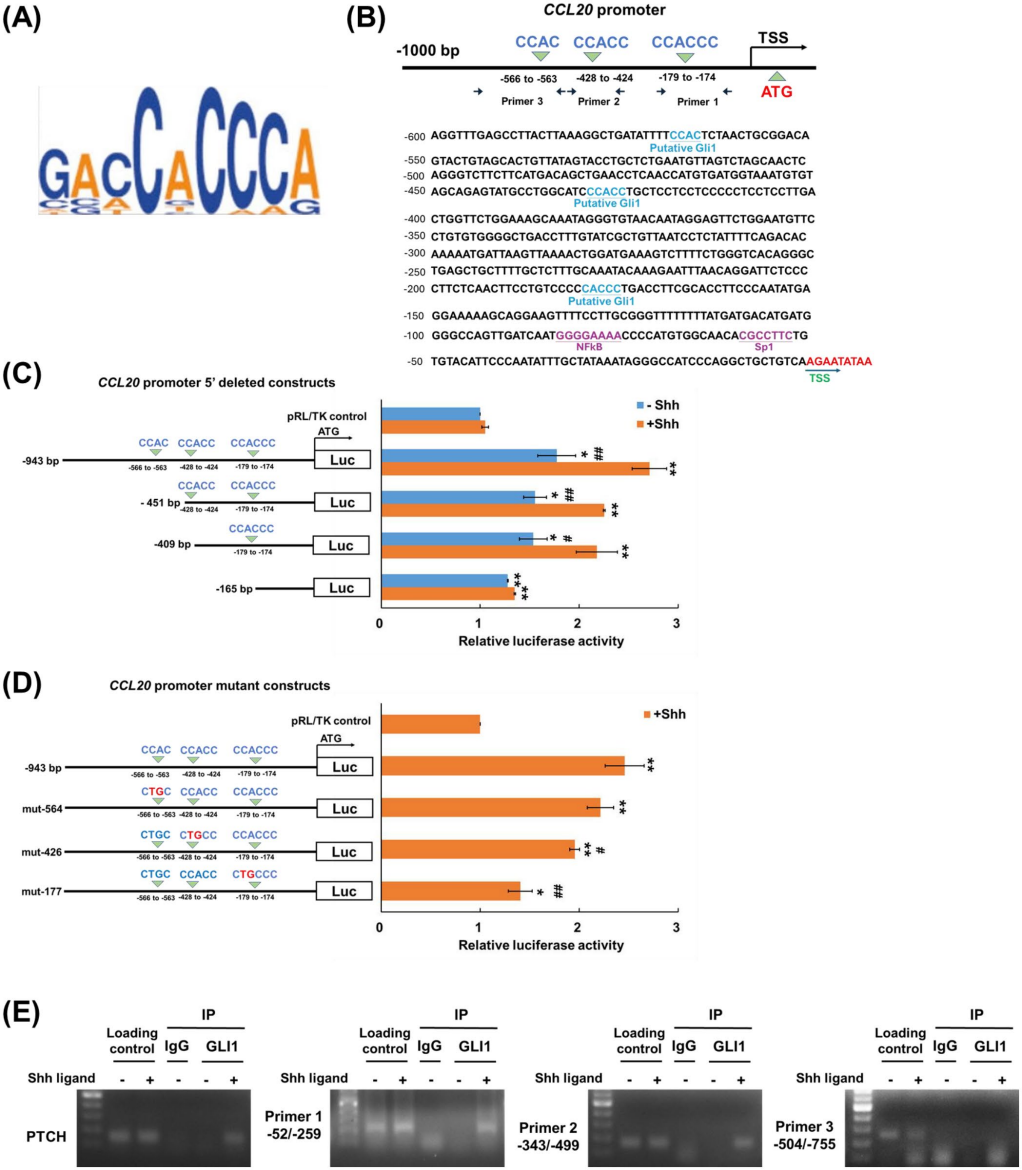
GLI1 transcriptionally regulates the expression of CCL20. (A) The consensus sequence of the GLI1 binding site. (B) Prediction of GLI1 binding sites on −1000 to −1 bp of the CCL20 promoter region. Blue indicates the GLI1 consensus sequence. Arrows indicate the primers used in the ChIP assay. The lower panel shows the sequence of the CCL20 promoter region and putative transcription factor binding sites. (C) The luciferase reporter assay with the CCL20 5′ deleted promoter region. (D) The luciferase reporter assay with the CCL20 mutated promoter region. (E) ChIP assay for GLI1‐binding sites in the CCL20 gene promoter in PLC5 cells. After chromatin immunoprecipitation by anti‐GLI1 antibody, CCL20 promoter binding by GLI1 was amplified by PCR with indicated primers. IgG was used as the negative control. Results are expressed as the mean ± SD of three separate experiments (*p < 0.05, **p < 0.01, vs. vector controls; ^#^ *p* < 0.05, ^##^ *p* < 0.01, vs. Shh treatment).

### 3.4 GLI1 Transcriptionally Regulates CCL20 Expression

As a transcription factor, GLI recognises a consensus binding sequence, GACCACCCA, where the middle CACC is critical for binding (Figure 4A) [30]. To identify the putative GLI1 binding sites on the *CCL20* gene, we searched for the consensus sequences upstream of the transcription start site (TSS), specifically from −1000 to −1 bp, corresponding to chr2:227805739 to 227817564 on the human GRCh38 Assembly (hg38). Three putative GLI1 binding sites: CACCCC sequence at −174 to −179 bp, the CCACC sequence at −424 to −428 bp and CCAC sequence from −563 to −566 bp, were identified (Figure 4B). The −943 to −1 bp sequence from the TSS of the *CCL20* gene was cloned into a luciferase reporter construct (pRL‐TK) and a series of 5′ deletions were generated to determine the region responsible for promoter activity in luciferase expression. PLC5 cells were transfected with either control or CCL20 promoter constructs, and promoter activity was analysed with or without Shh ligand treatment. All 5′ deleted *CCL20* promoter constructs showed a slight increase in activity compared to the vector control without Shh ligand (Figure 4C). Notably, the full‐length −943/−1 construct and truncated constructs containing −451/−1 or −409/−1, which include putative GLI1 binding sites, significantly increased promoter activity upon Shh treatment, whereas the −165/−1 fragment, lacking GLI1 binding sites, showed no difference with or without Shh treatment. This indicates that GLI1 promotes transcription within the −945 to −165 bp region (Figure 4C). To confirm specific GLI1 binding sites, mutant constructs with CA to TG substitutions in the consensus sequences were generated (Figure 4D). In response to Shh ligand, both mut‐177 and mut‐426 mutants showed significant reductions in promoter activity compared to the wild‐type construct, while mut‐564 did not (Figure 4D). This suggests two key GLI1 binding regions, at −174 to −179 bp and −424 to −428 bp. The mut‐177 construct nearly abolished promoter activity to the vector control level, indicating that the −174 to −179 bp region may be the primary GLI1 binding site (Figure 4D).

To demonstrate direct GLI1 binding to the *CCL20* promoter in PLC5 cells, we performed chromatin immunoprecipitation (ChIP) assays using an antibody against GLI1. PTCH, a known direct transcription target of GLI1, served as a positive control [31], with IgG as the negative control. Three primer pairs were designed to cover the putative GLI1‐binding sites on the *CCL20* promoter (Figure 4B). The ChIP assay revealed that GLI1 interacts with multiple segments of the *CCL20* promoter in response to Shh ligand treatment. Primer pairs 1 and 2 yielded positive results, while Primer 3 did not (Figure 4E). Consistent with the luciferase reporter assay, the ChIP analysis indicates that GLI1 binds directly to the *CCL20* promoter within the −174 to −179 bp and −424 to −428 bp regions upon Hh pathway activation.

**FIGURE 5:**
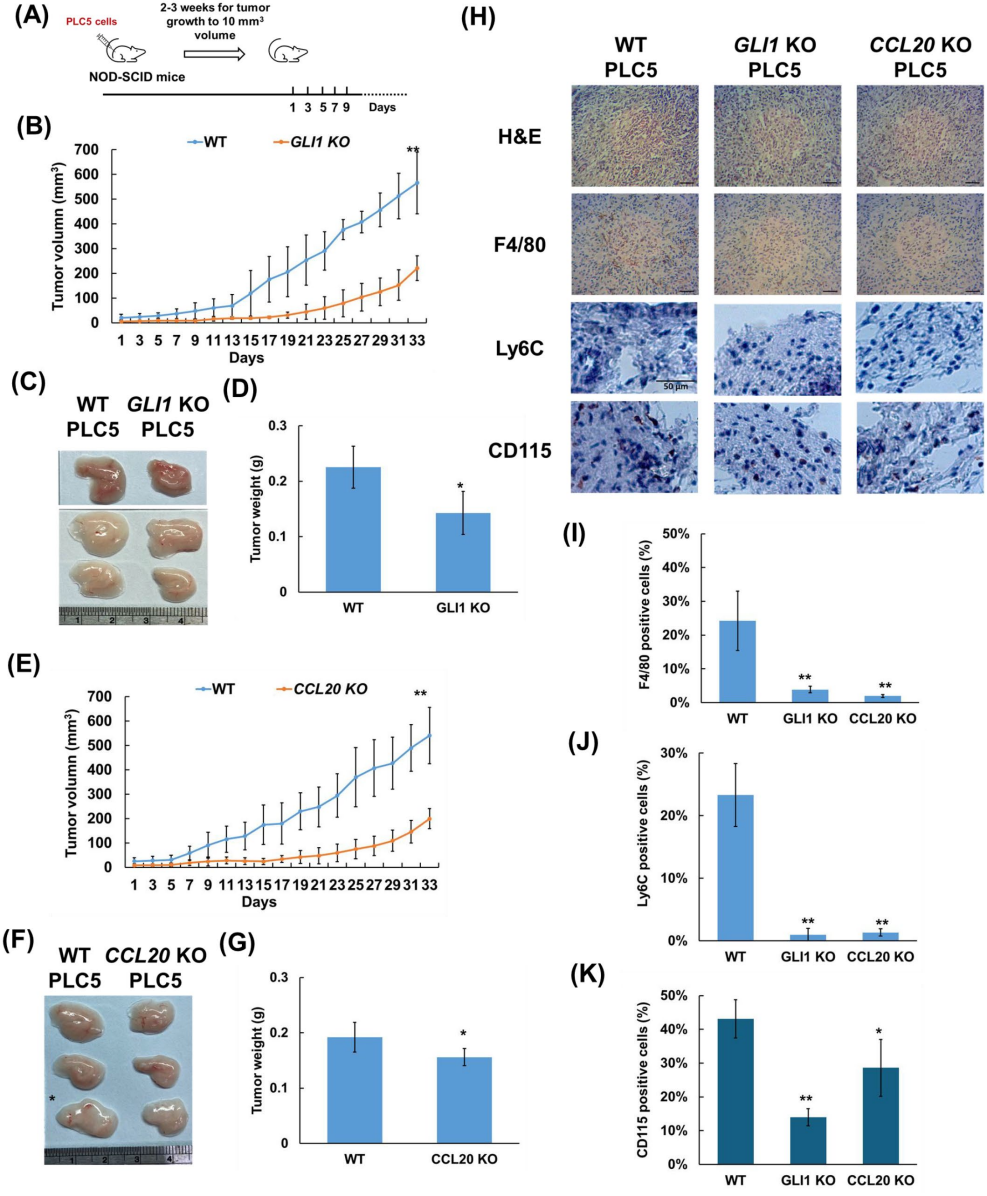
GLI1 and CCL20 knockout suppress tumourigenesis and monocyte infiltration in vivo on the PLC5 xenograft mouse model. (A) Scheme of PLC5 tumour cell engraftment in NOD/SCID mice. (B) Tumour growth curve of WT, *GLI1* KO PLC5‐bearing mice (*n* = 5). (C) Representative images of xenograft tumours formed in mice that were injected with WT or *GLI1* KO PLC5 cells. (D) The weight of the WT and GLI1 KO PLC5 xenograft tumours isolated from mice. (E) Tumour growth curve of WT, *CCL20* KO PLC5‐bearing mice (*n* = 6). (F) Representative images of xenograft tumours formed in mice that were injected with WT or *CCL20* KO PLC5 cells. (G) The weight of the WT and CCL20 KO PLC5 xenograft tumours isolated from mice. (H) Representative images of H&E staining, F4/80, Ly6C and CD115 IHC staining and the quantification of (I) F4/80, (J) Ly6C and (K) CD115 in WT, *GLI1* KO and *CCL20* KO PLC5 xenograft tumours derived from mice. The percentage of F4/80‐positive cells was quantified using ImageJ. Scale bar is 100 μm for H&E and F4/80 and 50 μm for Ly6C and CD115. Results are expressed as the mean ± SD (*p < 0.05, **p < 0.01, vs. WT cells).

### 3.5 GLI1 or CCL20 Knockout Decreases Tumour Growth and Monocyte Infiltration In Vivo

To investigate the impact of GLI1 and CCL20 on HCC progression and monocyte infiltration in vivo, we established PLC5 cell lines with homozygous knockout (KO) of *GLI1 or CCL20* using the CRISPR/Cas9 technique. The characterisation of *GLI1* KO and *CCL20* KO PLC5 cells is shown in Figures S1 and S2, respectively. The effect of GLI1 and CCL20 on tumour growth and monocyte infiltration was evaluated using a xenograft mouse model. The NOD‐SCID mice were transplanted with wild‐type (WT) PLC5 cells, *GLI1* KO or *CCL20* KO PLC5 cells, and the tumour growth was monitored over time (Figure 5A). Tumour growth in mice was inhibited in mice implanted with *GLI1* KO or *CCL20* KO cells compared to those with WT cells (Figure 5B,E). At the end of the animal experiment, the mice were sacrificed, and tumours bearing *GLI1* KO or *CCL20* KO cells exhibited reduced size and weight relative to WT controls (Figure 5C,D,F,G). Additionally, IHC staining revealed decreased percentages of monocyte/macrophages, which are detected by F4/80, Ly6C or CD115 markers, in tumours with *GLI1* KO or *CCL20* KO cells compared to WT tumour cells (Figure 5H–K). These results indicate that the Hh pathway and CCL20 contribute to monocyte recruitment in vivo. However, since CSF1, a known target of the Hh pathway, can induce F4/80 expression in macrophages, we cannot exclude the possibility that the reduction in F4/80‐positive cells observed in *GLI1* KO PLC5 tumour may be partially due to downregulation of CSF1 expression.

### 3.6 CCL20 Expression Is Positively Correlated With GLI1 Expression and Indicates Poor Survival in HCC Patients

**FIGURE 6:**
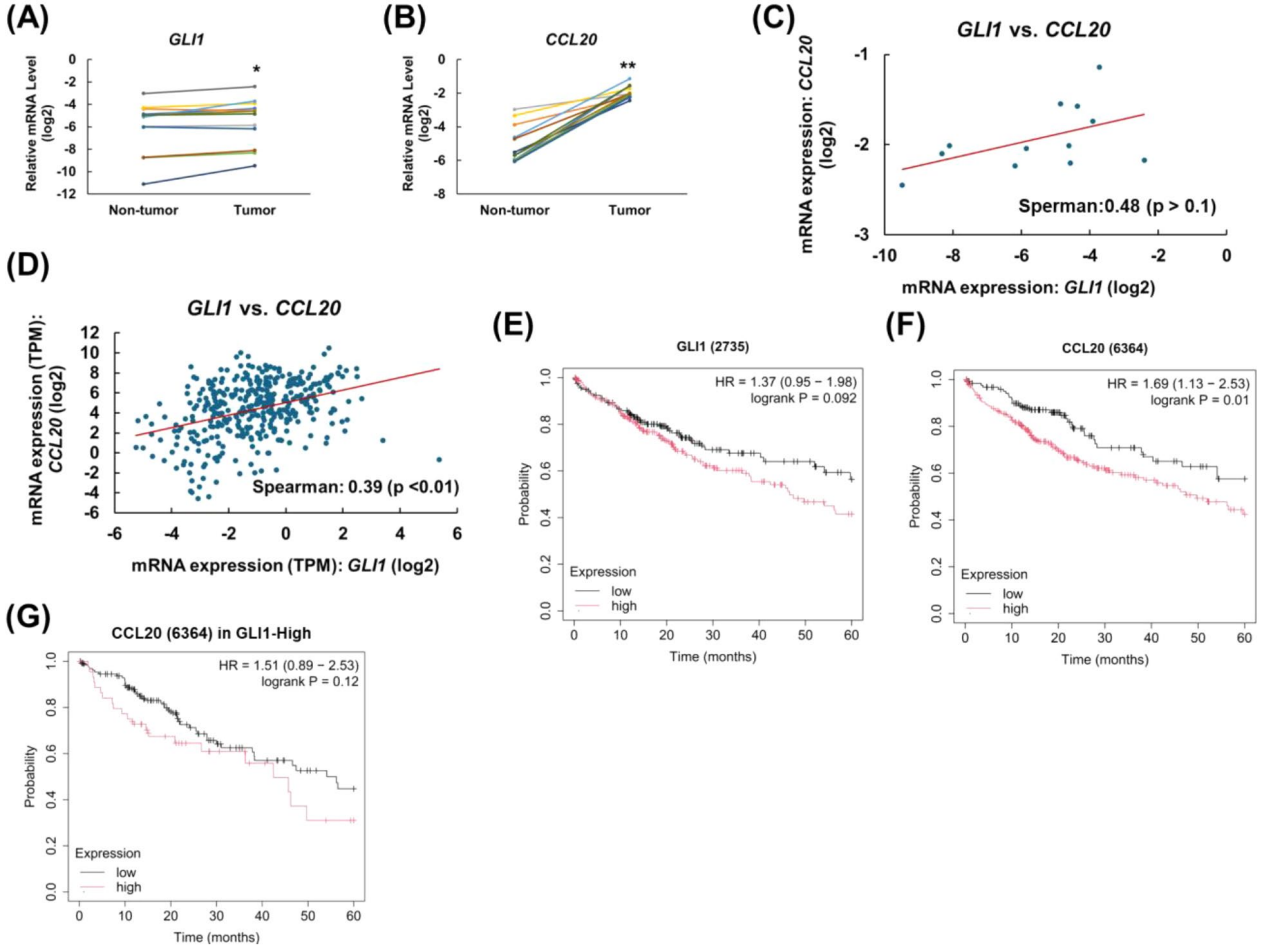
The correlation of GLI1 and CCL20 expression in clinical HCC samples. The mRNA expression of (A) GLI1 and (B) CCL20 in HCC tumour and adjacent non‐tumour liver tissues from 12 HCC patients. A paired *t*‐test was used for statistical analysis. (C) Correlation between the expression of *GLI1* and *CCL20* mRNA in HCC patients. Data are presented as each value. (D) Analysis of the Hepatocellular Carcinoma TCGA database (TCGA, GDC) (*n* = 329) using cBioPortal showing the correlation between GLI1 and CCL20 mRNA levels. The prognostic effect of *GLI1* and *CCL20* mRNA expression in www.kmplot.com. Kaplan–Meier curve in liver cancer patients (*n* = 364) for (E) GLI1 and (F) CCL20 was plotted. Auto select best cutoff value of gene expression was chosen to split the patient samples into two groups and the follow‐up threshold was set as 60 months. Red indicates the overall survival (OS) curve of the patient with a higher mRNA expression; the black line indicates the OS curve of the patient with a lower mRNA expression. (G) Prognostic effect of *CCL20* mRNA expression in the GLI1‐high subgroup (*n* = 177) from (E). (*p < 0.05, **p < 0.01).

To address the clinical association of GLI1 and CCL20 in HCC, the expression levels of *GLI1* and *CCL20* mRNA in samples from the tumour and paired adjacent nontumour tissues of 12 HCC patients were examined. mRNA levels of *GLI1* or *CCL20* were found to be significantly elevated in tumour tissues compared to paired adjacent non‐tumour liver tissues (Figure 6A,B). Despite this, no significant correlation was observed between *GLI1* and *CCL20* expression in the tumour samples due to the limited sample size (Figure 6C). To explore this potential association further, a larger dataset of 329 HCC samples from the TCGA database (Hepatocellular Carcinoma: TCGA, GDC) was analysed using the cBioPortal platform (Figure 6D). This analysis revealed a positive correlation between *CCL20* and *GLI1* expressions (*r* = 0.39, *p* < 0.01) via Spearman correlation, suggesting that *GLI1* and *CCL20* may be functionally linked in HCC tumours. Additionally, to investigate the impact of GLI1 and CCL20 on patient prognosis, survival analysis was conducted on 364 HCC patients using the Kaplan–Meier Plotter database [32]. The results of the analysis showed that high expression of either GLI1 or CCL20 was associated with significantly decreased overall survival (OS) (Figure 6E,F), implying that CCL20 may function as an oncoprotein in HCC. Furthermore, in GLI1‐high patients, elevated *CCL20* mRNA expression predicted worse overall survival compared with low *CCL20* expression (Figure 6G), indicating the potential of GLI1 and CCL20 as combined prognostic biomarkers.

## 4 Discussion

The Hh signalling pathway plays a significant role in various biological processes, including cellular proliferation, stemness and survival [33]. While its normal function is essential for organ development, abnormal activation of the Hh signalling pathway has been linked to numerous cancers, such as glioma, pancreas cancer, breast cancer, basal cell carcinoma and medulloblastoma [34, 35]. Recently, the Hh pathway has been extensively studied in HCC [36, 37, 38]. Hh signalling has no function in the adult liver, and normal hepatocytes display undetectable levels of Hh signal proteins [17, 38]. It has been shown that Shh ligands have been identified in approximately 60% of HCC tumour tissues, and high expressions of *PTCH‐1* and *GLI1* mRNA have also been detected in HCC tissues. It has been demonstrated that the hepatic expression of Shh promotes the activation of hepatic stellate cells to induce liver fibrosis [22]. Moreover, the activation of GLI increases osteopontin (OPN) to promote a macrophage‐mediated proinflammatory response in a mice model of nonalcoholic fatty liver disease (NAFLD), and Hh signalling promotes M2 polarisation of tumour‐associated macrophages for an immunosuppressive environment, suggesting the Hh pathway is involved in modulating the inflammatory microenvironment [39, 40].

It has been shown that *GLI1* may directly target the production of certain inflammatory cytokines and chemokines, such as *IL6*, *IL8*, *CCL2* and *CSF1*, while others like *TNF*, *IL1B*, *IL12*, *IL17* and *IL23* are regulated through the Hh signalling pathway. RNA expression analysis of HCC cells treated with or without Hh pathway inhibitor HPI‐1, followed by NSG analysis, highlighted DEGs associated with HSC activation, cytokine storms, and the tumour microenvironment, all suggesting that Hh signalling modulates the HCC tumour microenvironment. According to Ingenuity Pathway Analysis, the top 10 canonical pathways involving CCL20, IL17A, IL6 and CSF2 may serve as critical targets for Hh signalling in HCC. Furthermore, both IL6 and CCL20 are implicated in promoting monocyte migration, a key factor in the inflammatory tumour environment. While IL6 is a well‐established direct target of GLI1 and is known for its role in HCC progression [41], this study focuses on CCL20 as an Hh‐regulated chemokine, underscoring its potential role in influencing HCC pathology.

CCL20, also known as macrophage inflammatory protein‐3 alpha (MIP‐3α), is a cytokine with a critical role in immune regulation [42]. It is primarily involved in attracting immune cells to specific tissues, a process known as chemotaxis. CCL20 binds specifically to its receptor, CCR6, which is expressed on various immune cells, including monocytes, neutrophils, eosinophils, NK cells, T lymphocytes, B lymphocytes and dendritic cells [43]. The CCL20–CCR6 axis is implicated not only in inflammatory and infectious diseases but also in cancer progression. In several cancer types, including HCC, colorectal, breast, pancreatic, cervical and kidney cancers, this axis contributes to tumour microenvironment remodelling that supports cancer cell migration and proliferation [44]. Specifically, in HCC, patients with elevated CCL20 expression show poor recurrence‐free and overall survival rates. HCC‐mediated CCL20 production has been shown to recruit CCR6^+^CD5^+^ B cells, promoting angiogenesis and thus facilitating tumour growth. Blocking CCL20 has been found to inhibit angiogenesis, demonstrating its role in tumour vascular development [45]. Our findings show that activation of the Hh pathway upregulated the expression of CCL20, promoting monocyte migration. However, the use of NOD‐SCID mice in the xenograft model limits our ability to assess whether CCL20 inhibition affects other immune cell populations within the tumour microenvironment. This establishes the association between the Hh pathway and the CCL20/CCR6 axis in HCC progression, highlighting a mechanism by which Hh pathway activation may drive tumour‐promoting inflammation in HCC.

The *CCL20* promoter region contains a nuclear factor (NF)‐κB binding site (between −105 and −91), an AP‐1 binding site (between −124 and +33), a specificity protein 1 (Sp1) binding site (between −52 to −58) and two proximal CCAAT/enhancer‐binding proteins binding sites (−716 to −724 and −734 to −748) [46]. Based on the luciferase reporter assay and ChIP analysis, we identified two direct binding sites for GLI1 (−174 to −179 and −424 to −428). Mutation of the consensus sequence of the GLI binding site at −174 to −179 bp led to the abolishing of the transcriptional activity by the reporter assay, indicating the region between −174 and −179 bp is critical for Hh pathway‐mediated CCL20 expression.

In this study, our functional assays primarily focus on monocyte recruitment, as monocytes are a major source of tumour‐associated macrophages that strongly shape HCC's immunosuppressive microenvironment. The Hh‐CCL20‐CCR6 axis may also influence other CCR6‐positive immune subsets and tumour cell‐intrinsic processes, including metastasis and angiogenesis. Given the complexity of chemokine networks, the Hh‐CCL20 axis appears crucial for monocyte recruitment, although the involvement of other chemokines cannot be excluded. The use of THP‐1 cells and NOD‐SCID mice is a limitation, as these models may not fully reflect the heterogeneity of primary monocytes or the complexity of immune interactions in HCC. While they provide a consistent platform for assessing CCL20–CCR6‐mediated monocyte recruitment, they may influence the magnitude and context of the observed effects. Future studies using primary monocytes and immune‐reconstituted models will be essential to validate these findings in a physiologically relevant setting.

**FIGURE 7:**
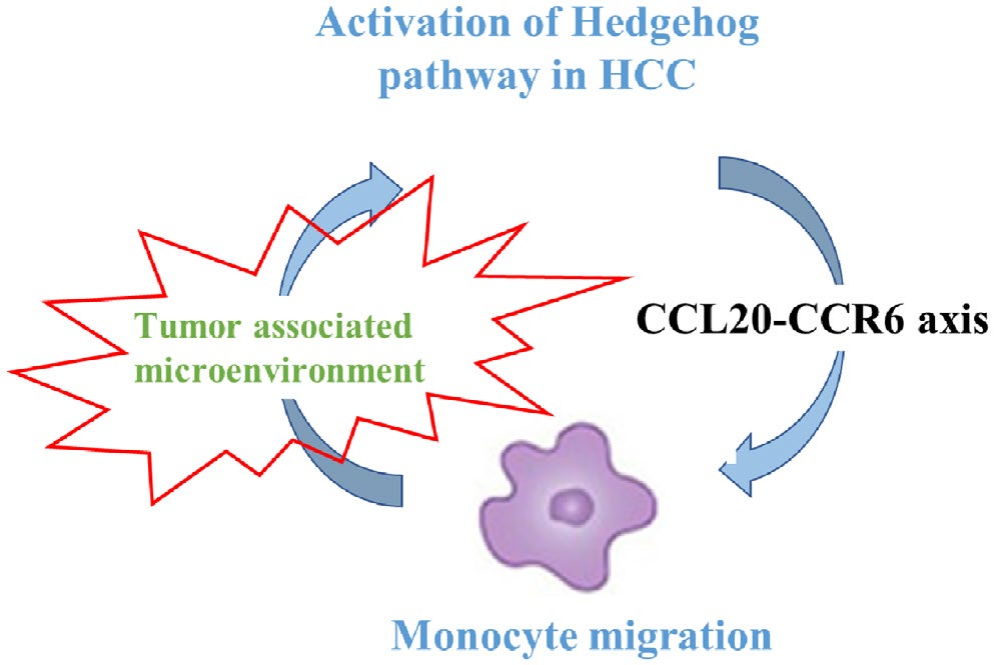
The Hedgehog pathway in hepatocellular carcinoma regulates monocyte infiltration through the CCL20–CCR6 axis for tumourigenesis.

In summary, the study demonstrated that the Hh pathway regulates CCL20 expression at the transcriptional level. The activation of the Hh pathway in HCC cells regulates CCL20 expression by binding to its promoter region via the downstream transcription factor GLI1. The released CCL20 recruits monocytes into the tumour microenvironment by interacting with its receptor, CCR6. This interplay between the Hh pathway and the CCL20–CCR6 axis modulates the tumour microenvironments, thereby promoting HCC progression (Figure 7). Given this mechanism, the abnormal activation of the Hh signalling pathway and the CCL20–CCR6 axis serve not only as a potential biomarker for HCC but also represent a promising therapeutic target to inhibit tumour growth and improve patient outcomes in HCC.

## Author Contributions

**Pei‐Han Chu:** data curation (lead), formal analysis (lead), investigation (lead), methodology (lead). **Yu‐Fu Hsu:** investigation (lead), methodology (supporting). **Chen‐Yi Chang:** data curation (supporting), formal analysis (supporting), investigation (supporting). **Chuen‐Miin Leu:** conceptualization (supporting), project administration (supporting). **Kuo‐Hsin Chen:** resources (lead). **Chiung‐Fang Chang:** conceptualization (lead), funding acquisition (equal), project administration (equal), supervision (equal), writing – review and editing (equal). **Ping‐Hui Tseng:** conceptualization (equal), formal analysis (lead), funding acquisition (equal), project administration (equal), supervision (lead), validation (lead), writing – original draft (lead), writing – review and editing (equal).

## Conflicts of Interest

The authors declare no conflicts of interest.

## Supporting information


**Tables S1–S2:** jcmm70824‐sup‐0001‐TableS1‐S2.pdf.

**Figures S1–S2:** jcmm70824‐sup‐0002‐FigureS1‐S2.pdf.

## Data Availability

The data that support the findings of this study are available from the corresponding author, P.‐H.T., upon reasonable request.
