# Supplementary material for: The Hedgehog Pathway Promotes Monocytes Infiltration Through CCL20–CCR6 Axis in Hepatocellular Carcinoma
Source: J Cell Mol Med. 2025 Sep 5;29(17):e70824. doi: 10.1111/jcmm.70824 (PMC12413313; doi:10.1111/jcmm.70824)
Supplement: Supplementary file 1 — Tables S1–S2: jcmm70824‐sup‐0001‐TableS1‐S2.pdf. [file JCMM-29-e70824-s001.pdf]

Table S1

Real-time PCR primer sequences

Gene	Forward primer (5'-3')	Reverse primer (5'-3')
GLI1	AGCGTGAGCCTGAATCTGTG	CAGCATGTACTGGGCTTTGAA
IL6	GACCCAACCACAAATGCCA	GTCATGTCCTGCAGCCACTG
CCL20	ATGTGCTGTACCAAGAGTTTGAC	CCAATTCCATTCCAGAAAAGCC
CSF2	ACCTGCCTACAGACCCGCCT	GAAGTTTCCGGGGTTGGAGGGC
IL17	AACGCCGAGGCCAATAACTTTC	AGGGTCCTCATTGCGGCTCAGA
CCR6	TGTCACAGAAGTCCTGGCTTTC	TACTTCCTTCTCACACACCACAG
IL1B	ACGAATCTCCGACCACCACT	CCATGGCCACAACAACTGAC
IL10	GATGCCTTCAGCAGAGTGAA	CCCAGGTAACCCTTAAAGTCC
ARG1	TCCAAGGTCTGTGGAAAAG	ATTGCCAAACTGTGGTCTCC
GAPDH	GTCATCATATTTGGCAGGTT	GAAGGACTCATGACCACAGT

sgRNA target sequences

Gene	Target sequence
gGLI1	TGTGGTCCCCATGACTCTGC
gCCL20	GTGCTGCTACTCCACCTCTG

ChIP assay target primers

Target	Forward primer (5'-3')	Reverse primer (5'-3')
PTCH1	CGAAGCCGAGGATGCACACACT	TTGGGTTTCTGCGACGCGATTG
Primer 1	GGTCACAGGGCTGAGCTGCTT	AGAAGGCGTGTTGCCACATGG
Primer 2	CAGGGTCTTCTTCATGACAGC	CACAGGAACATTCCAGAACTCC
Primer 3	CCTCAACAATTCTGAGGCTC	GCTAGACTAACATTCAGAGCAG

Table S2 Differential expressed genes in canconical pathway analysis

Ingenuity Canonical Pathways	-log(p-value)	Ratio	z-score	Molecules
Pathogen Induced Cytokine Storm Signaling Pathway	14.5	0.171	-4.464	BHLHE40,CASP1,**CCL20**,CLCF1,COL12A1,COL16A1,COL1A1,COL1A2,COL3A1,COL4A1,COL4A5,COL5A1,COL5A2,COL5A3,COL6A1,COL6A2,COL6A3,COL7A1,COL8A1,CSF2,CXCL1,CXCL12,CXCL2,CXCL3,CXCL5,CXCL6,CXCL8,FOS,FTH1,FTL,HLA-DRB1,HLA-DRB5,HMGCR,IFNAR1,IL11,IL1A,IL1B,IL1RAP,**IL6**,IL6ST,LIF,MAPK3,MYC,PYCARD,SOCS3,SRGN,STAT3,STING1,STXBP2,TGFB1,TGFB2,TNFRSF11B,TNFRSF1B,TNFSF12,TNFSF4,VEGFA,VEGFB,VEGFC
Tumor Microenvironment Pathway	14.3	0.225	-2.082	ARAF,CD274,CD44,COL1A1,COL1A2,COL3A1,**CSF2**,CSF3,CXCL12,CXCL8,FAS,FGF1,FGF2,FN1,FOS,ICAM1,IGF2,IL1B,**IL6**,ITGA5,ITGB3,MAPK3,MMP1,MMP14,MMP2,MMP3,MYC,NRAS,PDCD1LG2,PIK3R2,PTGS2,RALA,STAT3,TGFB1,TGFB2,TNC,TSLP,VEGFA,VEGFB,VEGFC
IL-17 Signaling	9.44	0.184	-3.656	**CCL20**,CLCF1,**CSF2**,CSF3,CXCL1,CXCL3,CXCL5,CXCL8,FOS,HSP90AA1,HSP90B1,IL11,IL1A,IL1B,**IL6**,LIF,MAP2K3,MAPK3,MMP2,MMP3,NRAS,PIK3R2,PTGS2,RALA,TGFB1,TGFB2,TNFRSF11B,TNFSF12,TNFSF4,TRAF3IP2,VEGFA,VEGFB,VEGFC
