# Supplementary material for: The Hedgehog Pathway Promotes Monocytes Infiltration Through CCL20–CCR6 Axis in Hepatocellular Carcinoma
Source: J Cell Mol Med. 2025 Sep 5;29(17):e70824. doi: 10.1111/jcmm.70824 (PMC12413313; doi:10.1111/jcmm.70824)
Supplement: Supplementary file 2 — Figures S1–S2: jcmm70824‐sup‐0002‐FigureS1‐S2.pdf. [file JCMM-29-e70824-s002.pdf]

Supplementary Figure 1

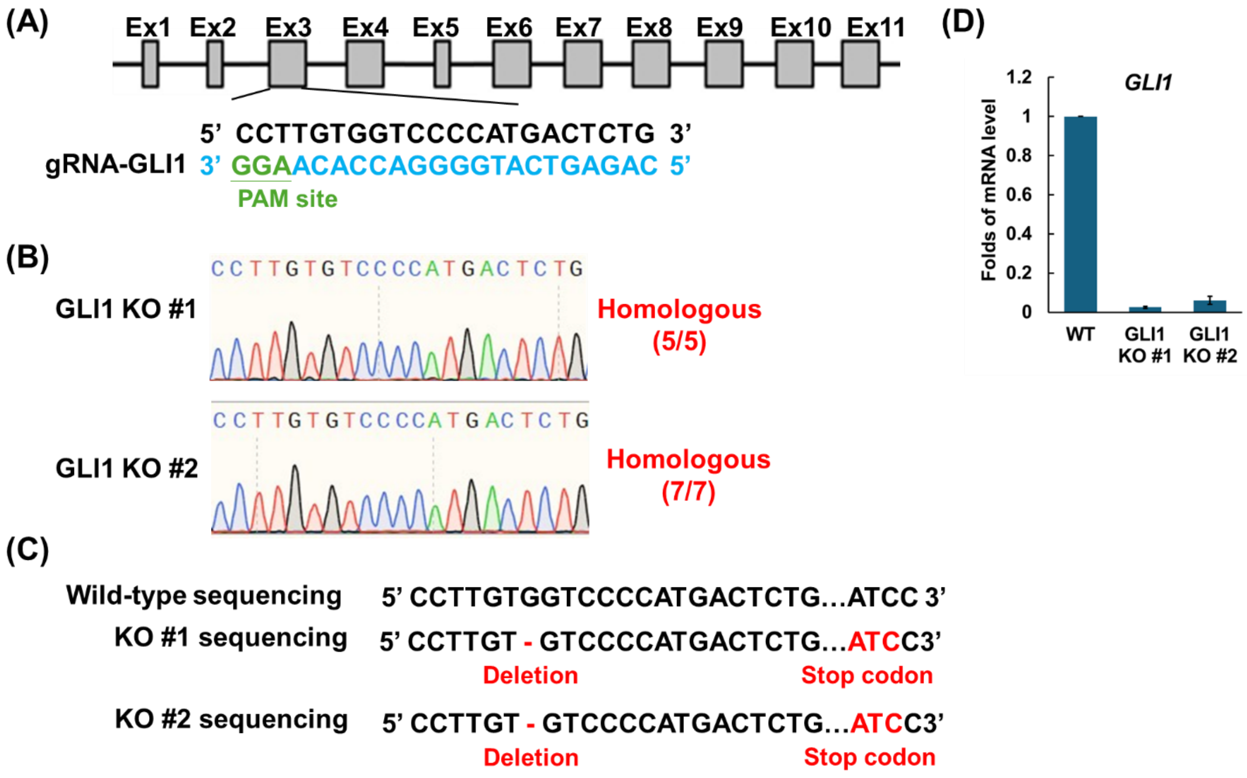

Supplementary Figure 1. Validation of *GLI1* knockout in PLC5 cells by using CRISPR/Cas9 system. (A) Target sequence of *GLI1* gene using Cas9/gRNA system. gRNA-targeting sequence is highlighted in blue and PAM sequence is highlighted in green. (B) Representative Sanger sequencing for *GLI1* gene in *GLI1* KO PLC5 cells. Two single colonies of *GLI1* KO PLC5 cells were picked up for sequencing. (C) The aliment of mutations in *GLI1* KO PLC5 cells with wild-type *GLI1* gene. (D) qPCR analysis of *GLI1* mRNA level in WT and GLI1 KO PLC5 cells.

Supplementary Figure 2

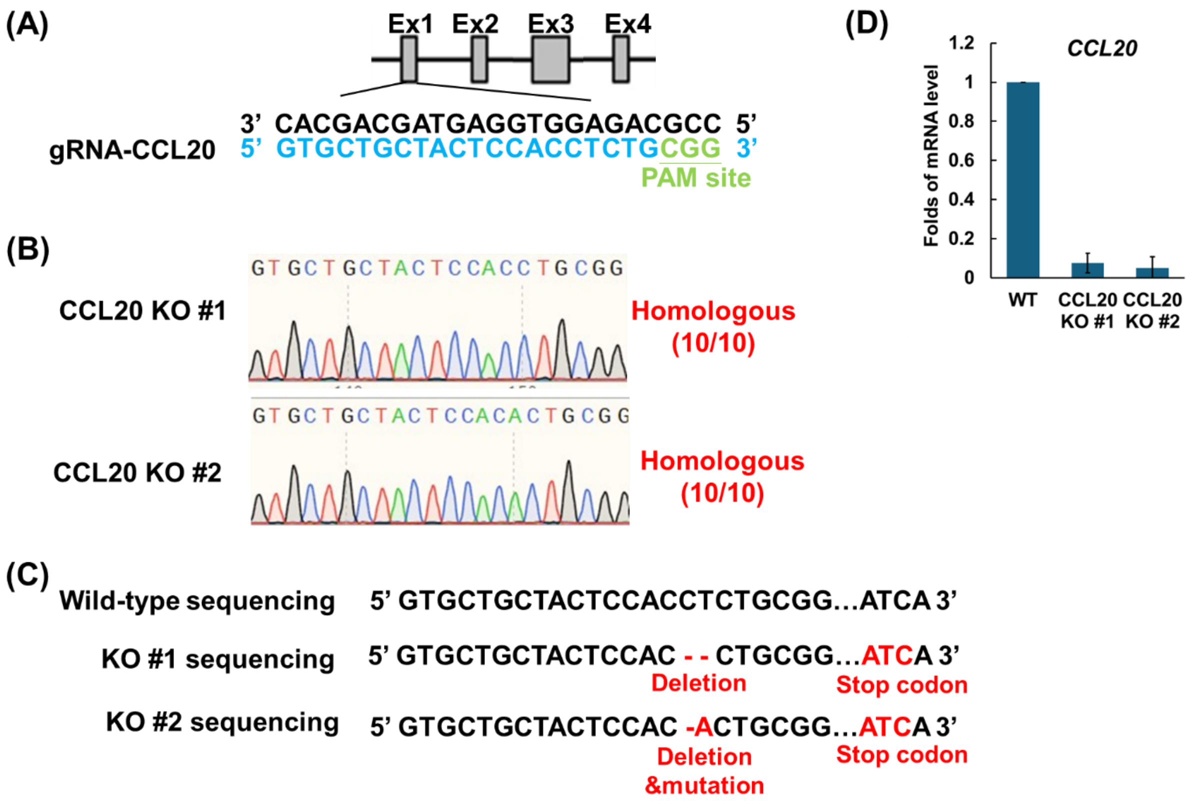

Supplementary Figure 2. Validation of *CCL20* knockout in PLC5 cells by using CRISPR/Cas9 system. (A) Target sequence of *CCL20* gene using Cas9/gRNA system. gRNA-targeting sequence is highlighted in blue and PAM sequence is highlighted in green. (B) Representative Sanger sequencing for *CCL20* gene in *CCL20* KO PLC5 cells. Two single colonies of *CCL20* KO PLC5 cells were picked up for sequencing. (C) The aliment of mutations in *CCL20* KO PLC5 cells with wild-type *CCL20* gene. (D) qPCR analysis of *CCL20* mRNA level in WT and *CCL20* KO PLC5 cells.
